# Lectures on Diseases of the Eye

**Published:** 1863-07

**Authors:** Henry D. Noyes

**Affiliations:** Assistant-Surgeon New York Eye Infirmary


### LECTURES ON DISEASES OF THE EYE.

By HENRY D. NOYES, M.D., Assistant-Surgeon N. Y. Eye Infirmary.

### DISEASES OF THE LACHRYMAL APPARATUS.

*Diseases of the Lachrymal Apparatus.*—The lachrymal sac and nasal ducts are the parts of the derivative apparatus which are most frequently diseased. The primary affection is usually *catarrh* of the mucous membrane.

This may originate from inflammation of the eyelids or of the Schneiderian mucous membrane. It often occurs in young children of a scrofulous constitution simultaneously with catarrh of the nasal passages, and it may take place as an acute attack at any period of life. It more usually comes on gradually, its incipient stages being scarcely observed. The secretion of the mucous membrane, which is naturally thick and glairy, becomes more consistent and opaque; it accumulates in the sac, and gives rise to a slight fulness at the inner angle of the eye. Pressure causes the swelling to subside by pushing the mucus into the nostril or into the conjunctival sac. There will be slight congestion of the palpebral conjunctiva. The eye will be filled with tears, and, on exposure to the wind, they will flow copiously over the cheek. The mucous membrane of the sac and duct is swollen and spongy, and the calibre of the nasal duct is diminished. The valvular folds of the membrane become tumefied, and oppose a decided obstacle to the flow of fluid into the nasal fossa. Hence, before organic stricture has formed, pressure over the distended sac will often cause its contents to regurgitate upon the conjunctiva.

Soon, however, the infiltration of the mucous membrane assumes a more organized form, because this tissue is a fibro-mucous layer. Fibrous tissue appears in it, and constitutes a permanent stricture. Its situation may be at any point of the duct, or through its whole extent; its common seat is at the valvule which marks the beginning of the duct. The formation of such a stricture is a very slow process; and, during this time, the patient is more or less annoyed by catarrh of the lachrymal sac. The sac becomes more and more distended, and its lining membrane more and more deeply diseased, giving rise to an abundant secretion of muco-pus.

It may attain an extraordinary size,—a hazel-nut might, in many instances, be accommodated within it. Pressure on this tumor evacuates its contents chiefly through the puncta. It may be painless, and continue for a long time in a passive state. But suddenly a new phase presents itself: acute inflammation is set up, and abscess forms. This may occur at various stages of the malady, either before or after the lachrymal sac has become distended enough to deserve the name of mucocele. The abscess forms in the loose areolar tissue around the sac, and soon ulcerates into it. It produces great swelling of the integuments. Especially in the sulcus, below the lower lid, the skin is tense, red, and hot; pain is severe until the pus has diffused itself into the areolar tissue. The pus tends to run along the loose tissue of the lower lid. If left to itself, the skin finally ulcerates, and the matter is discharged. Relief follows, but this is too often only the beginning of sorrows. From the opening, both pus and tears escape, showing a communication with the lachrymal sac. An ulcerated opening is very liable to remain patent, and result in fistula lachrymalis. This becomes not only a disfigurement but a source of continual annoyance. A succession of abscesses may occur; or the opening may close, pus form again, and make for itself a new exit, the suppurative process continuing for weeks.

A lachrymal fistula is not always an offensive opening, filled with fungous granulations; it may heal so far as to leave but a minute, almost capillary, aperture. Even in this state, it gives great trouble. Tears flood the eye, the conjunctiva is inflamed, and use of the eye is almost impossible.

I need not attempt to describe these cases any further. They are of chronic character, and have no tendency towards cure. If the nasal duct is obstructed, and this need only be a partial obstruction, it is the sufficient cause of a long train of annoyances, and of liability to intercurrent attacks of inflammation. Sometimes the nasal duct becomes totally occluded; it may be filled by bony tissue. The mucous membrane may be converted into pyogenic membrane. The lachrymal or maxillary bones may become carious. St. Yves speaks of operating for caries of the bones at the bottom of the orbit, and of the danger of destroying the eye in attempting to cure lachrymal disease.

*Treatment.*—In the early stages of catarrh of the lachrymal mucous membrane, the disease is easily managed. Astringent or caustic lotions are often applied to the eyelids. They allay the accompanying conjunctival inflammation. The very small quantum which enters the puncta can hardly be said to have a curative power over the lachrymal mucous membrane. To produce an effect upon this surface, an astringent wash must be injected through the canaliculi by Anel's syringe. The passages may first be washed out with warm water, and then the astringent introduced. In more chronic catarrh, and where the spongy mucous membrane partly closes the nasal duct, a weak solution of nitrate of silver may be injected, using it from five to ten grains to the ounce. In injecting nitrate of silver solutions through a canaliculus, care must be taken to prevent regurgitation through the other canaliculus, by squeezing it with forceps whose blades are not toothed or too rough. If the fluid can be forced into the nose, and its subsequent injection becomes more and more easy, this proceeding may cure the disease. If, however, there be great difficulty in forcing the fluid into the nose, and if there be no improvement, after a few trials, the presumption is, that stricture of the nasal duct has begun. It always requires considerable force to use the fine-pointed syringe of Anel, and the normal resistence it offers must not be mistaken for resistence in the lachrymal passages.

Upon this presumption of nasal stricture, Mr. Bowman's method of treatment must be adopted: the inferior punctum and canaliculus should be laid open, and a probe inserted into the masal duct. It is best to begin with No. 3 or 4, as they are less liable to wound the lining membrane. The largest size should be passed that will enter easily. The probe may, the first time, be left *in situ* for twenty minutes; then the sac and duct may be syringed out. It will often be found, in recent cases, that the probe will need but a few introductions, at intervals of two or three days; and the astringent or caustic injection will speedily remove the lingering catarrh. On the other hand, in more protracted cases, the stricture will be found less yielding; it gives a grating sensation as the probe goes through it. The amount of muco-purulent secretion may be small; probe No. 3 or 4 may enter, but No. 5 not without violence. Such a case requires persevering dilitation. Advance from a smaller to a larger size must sometimes be made gradually, at other times rapidly. The probe may be introduced at intervals of one to three days, according to the sensibility of the parts, and may be left in place half an hour at a time. Even where the largest size has been reached, it should be passed a number of times, at intervals of a week, to prevent contraction of the stricture. There is much the same propensity to contraction in strictures of this canal as in strictures of the urethra. In fact, the philosophy of treatment in the two cases is identical. The time necessary to effect a cure is, of course, variable; it will be from one to six months.

The attempt is to restore the tissues to their healthy condition; and, to do it, the mechanical obstacle (the stricture,) must be overcome, and permanently, while the chronic inflammation of the mucous membrane is to be set aside. The mechanical treatment has a great influence in abating the chronic catarrh, because the irritating morbid secretions are thus set free. Often no other proceedings are necessary than passing the probe. Injections are needed only where there is profuse catharrhal secretion distending the lachrymal sac and irritating the conjunctiva.

A few words as to the manner of introducing probes:—Each end of the instrument has a different size; and the most convenient kind has a shield soldered to the middle, upon which the numbers are stamped, and which enables the probe to be handled more readily. The sizes were, originally, from one to six. I have found that larger probes can be passed, and have added Nos. 7 and 8. These large sizes require the canaliculus to be divided quite up to the lachrymal sac.

In using the probe, it must be curved to correspond with the configuration of the face. Simply bending it for half an inch from its tip is not enough, neither is the same curve adapted to all cases. The height of the nose and the prominence of the brow will greatly alter the depth and course of the lachrymal duct. The probe then must have a large curve, that is, it should be an arc of a circle, whose radius will be shorter or longer as the case requires. The larger sizes, from 5 to 8, must be of pure silver; the smaller sizes should be alloyed to give them stiffness. It is better to stand behind the patient, with his head resting against your person; tell him to look upwards; draw the lower lid downwards and outwards with one hand, and with the other use the probe. The concave side of the probe must be kept forwards, the point carried horizontally along the divided canaliculus until, entering the lachrymal sac, it strikes against the opposite bony wall. It is sometimes difficult to get thus far,—the mucous membrane of the canaliculus may be folded over the probe, or the passage may be too narrow. When this is the case, the skin of the lid will be wrinkled and pushed inwards as force is applied to the instrument. To avoid folds of the mucous membrane, it is well to make the point of the probe press forward as it is pushed along. If the passage be too narrow, it must be cut with the knife, or a smaller size taken. If the point strike the inner wall of the lachrymal sac, (and this will be recognized by its solid resistence,) it should be held fixed while the other end is brought to the vertical position.

When this change is made, and not until then, should the probe be pushed downwards. If it has been properly curved, it will not press against the brow in going down, nor meet any resistance, except from the stricture. But, if the probe be too straight, it presses painfully against the eyebrow, the point scrapes the mucous membrane of the nasal duct, and there will be great risk of making a false passage. In pushing the probe downwards, its point should be directed a little outwards towards the ala nasi. In following these rules strictly, as to direction and shape of the probe, considerable force may be safely employed, but no violent efforts should be made. If one probe will not pass readily, try a smaller size. When the probe is fully down, the shield comes opposite the eyebrow, and the probe points to the ala nasi. If the upper end pitches forwards, or the direction deviates from the above, a false passage has been made. This begins usually at the top of the nasal duct, and is made outside of the superior maxillary bone, under the tissues of the cheek. Fortunately, no serious harm is thus inflicted, if the mistake be not persisted in: the wound will heal in a few days, and the attempt may be repeated. Ecchymosis of the lid or cheek often betrays this error.

The intermittent dilatation by probes must be persevered in for weeks and months,—the intervals becoming longer as the tendency to relapse diminishes. Very satisfactory results are thus obtained. Patients sometimes get tired of repeated probing, and, when they have been on the point of giving up treatment, in disgust, I have resorted to another method of accomplishing dilatation of the stricture. I have taken a piece of lead-wire of the same size as the probe which can be passed, rounded one end to make it smooth, and pushed this into the nasal duct; the upper end is bent at an acute angle, and hangs over the edge of the lower lid. This I have left *in situ* for one day or three days, and, in one case, for three weeks, without producing irritation of the eye. The stricture was kept dilated, and the epiphora ceased. I should not employ this method except on patients whom you can see at any time; but a few trials of it have given me a favorable impression of its value in shortening the duration of treatment and in making the dilatation more permanent.

This process of dilatation sometimes needs to be continued with astringent applications to the mucous lining of the sac, by a fine syringe, or by collyria dropped into the eye. More frequently such treatment is needless; the catarrhal inflammation abates, *pari passu*, as the obstruction yields.

*Phlegmonous inflammation and abscess* may take place either with or without stricture of the duct. When first it threatens, it may be aborted by applying two to four leeches over the sac, and by the assidious use of iced compresses. If suppuration cannot be avoided, employ warm fomentations, and make an incision into the sac as early as possible. I would urge the importance of an early opening; if no pus appears, the tension of the tissues is relieved, and the bleeding is serviceable: when suppuration shall take place, it will not undermine the skin, as it is prone to do when left to its own course. Be not stinted in the size of your incision, and aim to penetrate the sac. The best mode of doing the operation is, to stand behind the patient, who will be on his back or sit in a low chair, and use a straight bistoury. Put the point as nearly over the middle of the tendo oculi as the swelling will enable you to judge, holding the handle perpendicular to the plane of the face and turned a little outwards; thurst the point quickly backwards, so as to strike the lachrymal bone, and immediately carry it downwards and outwards. If the patient's head suddenly starts up as the knife enters, his movement makes the cut larger, and aids your purpose.

An abscess which opens by ulceration, or which has been too sparingly incised, is apt to fill up again, and require repeated incision. Sometimes it will linger along in this way for two or three months. It is then apt to degenerate into fistula lachrymalis. Fistula is not so likely to occur when an abscess is opened early and sufficiently. The cutaneous orifice of a fistula may be concealed by a thin layer of cuticle, or it may be pouting with fungous granulations. It may, in time, cicatrize, and contract to a capillary opening. The course of the fistula is sometimes crooked, but there is, generally, no difficulty in passing a probe through it into the lachrymal sac. If the fistula be recent, it may be closed by cauterizing it once, in two or three days, with a pointed crayon of nitrate of silver. Such are made by Squibb. But, if the fistula be old, and, in every doubtful case, there must first be an exploration of the nasal duct and lachrymal sac, to decide upon their condition. If their calibre and lining membrane are or may be made normal, then try to close the fistula. If there be a stricture, dilate it with probes *per vias naturales*, slitting up the canaliculus: do not attempt to dilate stricture through the fistula. But if the nasal duct be the seat of an unconquerable stricture, or be closed by ossific growth; if the lachrymal sac be enormously dilated, and its mucous lining have become a mere pyogenic membrane; if there be caries of the adjacent bones; if fistula have lasted a long time; or if a patient cannot spare the time which may be needful to restore the passages to a healthy state,—another proceeding must be adopted. This is the obliteration of the lachrymal sac and upper portion of the nasal duct. Tavignot adopts the obliteration of the canaliculi alone, but I do not deem this sufficient, certainly not in bad cases. This proceeding was in use a hundred years ago,—it is described by St. Yves. In late years it has been revived by Desmarres, and it is now very generally adopted. But, you will ask me: if you totally occlude the sac and duct, what will become of the tears? Will not epiphora be more distressing than ever? Bear in mind that you have an incurable disease of the passages; they are in a state of perpetual inflammation; they keep up a chronic conjunctivitis, and reflect irritation upon the lachrymal gland. Hence, there is a constant hypersecretion of tears, as well as obstruction to their natural escape.

If you destroy the inflamed lachrymal mucous membrane, and shut up the cavity which is the seat of disease, you remove the cause which provokes chronic conjunctivitis and excessive lachrymal secretion. Soon the conjunctiva recovers a healthy state, and tears cease to flow more than to meet the physiological demand. The fluid for moistening the eye is, ordinarily, supplied by the conjunctiva, and a slight excess is evaporated. When in the house, or where nothing irritates the eye, a patient is entirely comfortable; but when exposed to wind or dust, and tears flow more freely, they must stand in the conjunctival sac, or overflow the cheek. In the latter case, a patient with obliterated lachrymal sac suffers inconvenience. But this is admitting what is true of a multitude of surgical operations: they do not restore the perfect performance of function, they only mitigate an evil. Resected joints are not so good as healthy joints, but they are far better than anchylosis.

But you will ask: Why not insert a style? Simply, because a style answers no better purpose than does occlusion of the sac. It is a foreign body, an unsightly object, and an annoying thing to wear.

Dupuytren's tubes are far worse than styles: they become impacted in the nasal duct, and, by causing absorption of adjacent bony walls, they sometimes travel far out of their intended place, and often provoke serious suppuration. They are utterly out of use. A similar but less amount of mischief attaches to the style as being a foreign body, while obliteration of the sac and duct accomplishes, at least, all that the style can.

Another objection may be made in the supposed deformity which such an operation must cause. A scar is left at the inner angle of the eye, which is sometimes sunken, but is always linear, and is never conspicuous. I have done the operation upon the lachrymal sacs of a young lady of seventeen years, without at all maring the beauty of her fair face.

The mucous membrane of the lachrymal passages may be destroyed in a variety of ways. The most elegant method is by the galvano-caustic,—but the apparatus is expensive, and very liable to get out of order. The actual cautery is used more frequently than any other proceeding; then a variety of potential cauteries are used, such as nitric acid, caustic potash, butter of antimony, chloride of zinc, and nitrate of silver. In the Infirmary, we resort usually to the hot iron. The cauteries are of various shapes, bulbous or pointed, and one is bent at an obtuse angle within two inches of the point, to enable it to be thurst down into the nasal duct without burning the skin of the brow. It also has a bulb for retaining its heat. The irons are heated most conveniently in a dentist's furnace-lamp. They should not have more than a very dull red heat; it is better that they should not be at all red, than be too hot.

It is always necessary to use an anæsthetic; and, for this operation, I prefer sulphuric ether. The sac is laid freely open from its uppermost part across the tendon of the orbicularis, down a little distance upon the cheek. The incision must be at least an inch long, and its lower end curve outwards a little. When the sac is fully exposed, wait for the bleeding to stop,—ice may be applied to save time. The operation is much delayed by the copious bleeding which always occurs from capillary vessels; I have attempted to check it by persulphate of iron, but was more embarrassed by the coagula than if I had not used it. It is better to trust to ice, and pressure, and spontaneous coagulation. When the wound is dry, have it stretched open by retractors. These may be sharp hooks to catch the skin, or leaden spatulæ. It is well to use one leaden spatula which will at the same time cover the eyeball from harm.

The cauteries are applied carefully to the whole of the sac, and to as much of the nasal duct as can be reached, until the mucous membrane is well blackened. It is also well to introduce a fine cautery into the canaliculi, but is not always necessary.

Sometimes the reaction from the operation is smart. I have seen acute conjunctivitis and chemosis follow; there will always be considerable swelling of the lids. But, generally, the inflammatory reaction is moderate. Compresses dipped in iced water are the proper dressing.

The cauterization may not have been thorough, and a small fistulous opening will, after three or four weeks, remain. Unless the cavity is totally obliterated, the operation will be fruitless. If there should be a remaining pocket, a bit of solid nitrate of silver may be pushed into it and left there. This will usually suffice.

The heated iron produces less reaction than nitric acid or caustic potash; it can be more carefully managed, and it is not so liable to cause superficial necrosis of the bony walls. But, in private practice, the actual cautery would be looked upon with horror, and you may have to employ nitric acid or potassa fusa.

Even solid nitrate of silver is said to be adequate: a piece is put into the sac and left to dissolve. After a week or two another piece is thrust in, and this is repeated until occlusion is obtained.

You need not be alarmed if the bony walls should be denuded, and superficial necrosis occur. The healing will be protracted, but I have never seen serious ill effects result.

The time required for a cure by the actual cautery is about four weeks. I can only repeat, that the operation affords great relief, that it has, in almost all cases, been gratifying to myself, and that, while some objections to it are unfounded, those which do lie against it are such as may be urged against many well-established surgical operations.

One remark remains to be added. In young children, you are often unable to employ operative treatment,—probes and occlusion of the sac are out of the question. You may effect much by giving them cod-liver oil, iodide of potassium, by using astringent collyria, and by invigorating the general health in every practicable way.

Adults are sometimes averse to operative interference. You can alleviate the annoyance of their complaint by showing them how to empty the distended sac and avoid irritating the eye. Teach them to press with their fingers or handkerchief upon the lachrymal sac, and absorb the fluid which regurgitates by simple pressure, without rubbing the eyelids. Friction of the eyelids is very irritating, while gentle pressure empties the sac, dries the eye, and gives relief. It is important to keep the sac empty,—accumulations of muco-pus aggravates the inflammation and irritates the conjunctiva.—*American Medical Times.*

